# Quantitation of Residual Host Cell DNA in Recombinant Adeno-Associated Virus Using Droplet Digital Polymerase Chain Reaction

**DOI:** 10.1089/hum.2023.006

**Published:** 2023-06-16

**Authors:** Kiyoko Higashiyama, Yuzhe Yuan, Noriko Hashiba, Kyoko Masumi-Koizumi, Keisuke Yusa, Kazuhisa Uchida

**Affiliations:** Graduate School of Science, Technology and Innovation, Kobe University, Kobe, Japan.

**Keywords:** recombinant adeno-associated virus, host cell DNA, 18S ribosomal RNA, droplet digital PCR, HEK293 cells

## Abstract

Recombinant adeno-associated virus (rAAV) is a viral vector commonly used in gene therapy. Residual host cell DNA is an impurity that has been associated with the risk of infection and oncogenicity. Thus, it needs to be monitored for quality control. We aimed to develop a droplet digital polymerase chain reaction (ddPCR) method targeting 18S ribosomal RNA (rRNA) genes to quantitate residual host cell DNA. The copy number of the 18S rRNA gene was determined using two sets of primer pairs for 116- and 247-bp amplicons sharing the C-terminus. For conversion of the copy number of the 18S rRNA gene into the mass concentration of genomic DNA, the accurate copy number of 18S rRNA genes in HEK293 genomic DNA was determined by comparison with copy numbers of three reference genes (*EIF5B*, *DCK*, and *HBB*). Results showed that 88.6–97.9% of HEK293 genomic DNA spiked into rAAV preparations was recovered. The ddPCR-based assay was applied to rAAV preparations to quantitate residual host cell DNA as an impurity. Our findings indicate that the assay can be used for the quantitation and size distribution of residual host cell DNA in rAAV products.

## INTRODUCTION

Gene therapy products developed using viral vectors have been used for treating rare and monogenic diseases without treatment options. Recombinant adeno-associated virus (rAAV) is a viral vector commonly used in gene therapy.^1^ Many ongoing clinical studies are investigating the use of gene therapy in the treatment of diseases or disorders such as Alzheimer's disease, Parkinson's disease, Batten disease, mucopolysaccharidosis, gangliosidosis, and cancer.^2–5^

Gene therapies based on rAAV have been approved for clinical therapy: Glybera for treating lipoprotein lipase deficiency,^6^ Luxturna for Leber's congenital amaurosis,^5^ and Zolgensma for spinal muscular atrophy type 1.^7^ In addition, Roctavian is used for severe hemophilia A, Upstaza for severe aromatic l-amino acid decarboxylase deficiency, and Hemgenix for hemophilia B; these drugs have been authorized in the European Union, and FDA has approved Hemgenix in 2022.

The quality and safety of rAAV products are crucial, and impurities need to be removed. However, impurities remain even after nuclease treatment during rAAV purification. Some DNA impurities in the final products of rAAV are nuclease resistant because they are encapsulated in capsids.^8,9^ They are derived from host production cells, plasmids used for transfection, and recombinant viruses when used^10–12^ for rAAV production.

Host cell DNA has been associated with the risk of infection (*e.g.,* retroviruses) and oncogenicity (*e.g.,* activated oncogenes). Therefore, monitoring the amount of residual host cell DNA is necessary for quality control.^13,14^ DNA fragmentation disrupts the integrity of oncogenes, infectious units such as integrated retroviruses, and other functional sequences. The risk of oncogenicity and infection associated with residual host cell DNA should be mitigated by decreasing the amount and size of DNA. According to guidelines, the amount of residual host cell DNA should not exceed 10 ng per administration dose.^13,15^

Several studies have described the quantitation of residual host cell DNA in rAAV or vaccines through quantitative polymerase chain reaction (qPCR)-based methods using the human 18S ribosomal RNA (rRNA) gene,^8,16,17^ albumin gene,^18^ or Alu sequence.^14^ André et al.^17^ evaluated DNA fragmentation by comparing two sizes of amplicons (123 and 254 bp) in 18S rRNA using qPCR. Compared with qPCR, droplet digital polymerase chain reaction (ddPCR) has higher precision and a lower coefficient of variation (CV) in absolute quantification.^19^

The objective of this study was to develop a ddPCR-based assay for evaluating residual host cell DNA content and size applicable to rAAV products. We similarly quantitated the residual host cell DNA concentration using two sets of two primers and a probe overlapping the C-terminus in 18S rRNA genes and converted the copy number to estimate the mass concentration of host cell DNA in rAAV. In this study, we report the feasibility of the ddPCR-based method using host cell DNA for rAAV production.

## MATERIALS AND METHODS

### Genomic DNA and rAAV preparations

Genomic DNA was extracted from HEK293 cells (CRL-1573; American Type Culture Collection, Manassas, VA) using the QIAamp DNA Blood Mini Kit (no. 51104; Qiagen, Hilden, Germany) according to the manufacturer's instructions. Purified rAAV, 1908_rAAV1-CMV-ZsGreen1, 1909_rAAV2-CMV-ZsGreen1, and 1910_rAAV5-CMV-ZsGreen1 were provided by the Manufacturing Technology Association of Biologics.

### Digestion of genomic DNA using restriction enzymes

For ddPCR of the 18S rRNA gene, genomic DNA of HEK293 cells (5 μg) was digested using 25 units of restriction enzyme (*Hae*III, *Bam*HI, or *Pst*I) in digestion buffer (50 mM potassium acetate, 20 mM Tris-acetate, 10 mM magnesium acetate, and 100 μg/mL bovine serum albumin, pH 7.9) in a final volume of 50 μL for 4 h at 37°C.

Digestion was terminated by incubation at 65°C for 20 min, and digestion was confirmed through the 4150 TapeStation System using Genomic DNA ScreenTape (no. 5067-5365; Agilent, Santa Clara, CA). The digested DNA was diluted with 0.05% Pluronic F68 in 10 mM tris(hydroxymethyl)aminomethane-HCl and 1 mM disodium ethylenediaminetetraacetic acid (EDTA) buffer (TE_PF_), pH 8.0.

### Primer and probe design

**Figure 1.**
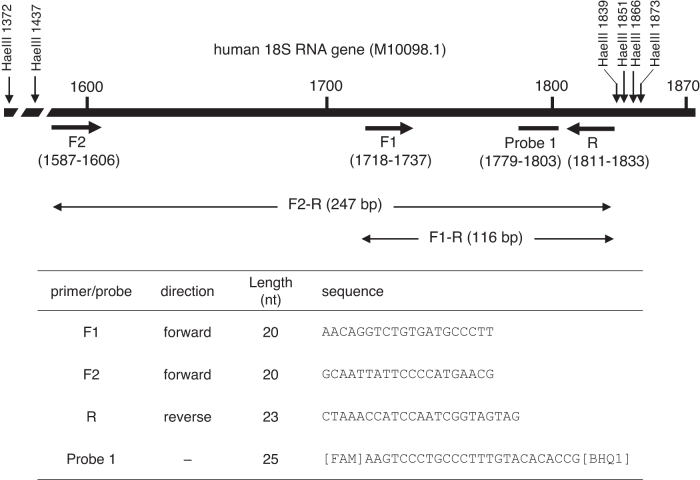
Location of primers and probe used for ddPCR in human 18S rRNA genes. The amplicons of 116 bp (F1-R primer pair) and 247 bp (F2-R primer pair) share the 116-bp C-terminus. ddPCR, droplet digital polymerase chain reaction; rRNA, ribosomal RNA.

All primers and probes were custom synthesized and high performance liquid chromatography purified (Eurofins Genomics K.K., Tokyo, Japan). The forward primer and probe for 18S rRNA were the same as those described by André et al.^17^ Two sets of primers were used to amplify overlapping fragments (116 and 247 bp) using the same internal probe and reverse primer (Fig. 1).

Primers and probes for three reference targets in the human genome (one copy/haploid) were as follows: eukaryotic translation initiation factor 5B (*EIF5B*), forward primer 5′-GCCAAACTTCAGCCTTCTCTTC-3′, reverse primer 5′-CTCTGGCAACATTTCACACTACA-3′, and probe 5′-[FAM]TCATGCAGTTGTCAGAAGCTG[BHQ1]-3′; deoxycytidine kinase (*DCK*), forward primer 5′-TGGTGGGAATGTTCTTCAGATGA-3′, reverse primer 5′-TCGACTGAGACAGGCATATGTT-3′, and probe 5′-[FAM]TGTATGAGAAACCTGAACGATGGT[BHQ1]-3′; and hemoglobin subunit beta (*HBB*), forward primer 5′-GCTGAGGGTTTGAAGTCCAACTC-3′ and reverse primer 5′-GGTCTAAGTGATGACAGCCGTACCT-3′.^20^ For rAAV-CMV-ZsGreen1 DNA, forward primer 5′-TTCGTGATCACCGGCGAGGGCAT-3′, reverse primer 5′-CCGTACATGAAGGCGGCGGACAA-3′, and probe [FAM]AACCTGTGCGTGGTGGAGGGCGGC[BHQ1] were used.

### ddPCR for three human reference genes, the 18S rRNA gene, and rAAV-ZsGreen1 DNA

The reaction mixture for ddPCR comprised 10 μL of ddPCR Supermix for probes (Bio-Rad Laboratories, Hercules, CA), 2 μL of primers (final concentration of each primer was 0.9 μM), 1 μL of probe (final concentration, 0.25 μM), and 1 μL of the template diluted with TE_PF_ in a final volume of 20 μL. Each DNA template was tested in triplicate. The plates were transferred to a QX200 Automated Droplet Generator (Bio-Rad). A 96-well plate containing the generated droplets was transferred to a C1000 Touch Thermal Cycler (Bio-Rad).

Cycling conditions were as follows: 10 min at 95°C, followed by 40 cycles of a two-step thermal profile of 30 s at 94°C and 60 s at 60°C. The plate was then transferred to a QX200 droplet reader (Bio-Rad), and data analysis was performed using the QuantaSoft software (version 1.7.4.0917; Bio-Rad). The threshold separating the negative and positive droplets was manually set just above the cluster of negative droplets or just below the cluster of positive droplets.

### Conversion of gene copy numbers determined by ddPCR into mass concentration

The mass concentration of nuclear DNA in the reaction mixture for ddPCR was calculated using the following equation:

(1) $${\left[gDNA\right]}_{conc}=\frac{n}{rF\left(6.02\times 10^{23}\right)}\times \left(3.28\times 10^{9}\right)\times 659.93\times 10^{12}pg∕\mu L$$

where *n* is the copy number of the gene per μL, *r* is the copy number of the target genes per HEK293 haploid genome equivalent (*r* = 1 for the reference genes), *F* is the dilution factor in the ddPCR mixture, 6.02 × 10^23^ is the Avogadro constant, 3.28 × 10^9^ is the number of nucleotides in the HEK293 haploid genome equivalent, and 659.93 is the average of the nucleotide pairs (C-G or A-T).^20^

According to the Genome Reference Consortium (GRCh38; https://www.ncbi.nlm.nih.gov/grc/human/data), the number of nucleotide pairs in the normal human female haploid is 3.01 × 10^9^ pairs. HEK293 cells were derived from the kidney of an aborted female human embryo,^21^ and cytogenetic analysis revealed that the structure of the genome was pseudotriploid.^21,22^ The average ploidy of the cell has been previously evaluated as 3.28,^22^ indicating that the equivalent ploidy in HEK293 cells is 1.09-fold higher than that of the human female haploid. In this study, we used 3.28 × 10^9^ ( = 1.09 × 3.01 × 10^9^) as the number of nucleotide pairs in HEK293 cells.

### Purification of rAAV

For rAAV production, 293T cells (CRL-3216; American Type Culture Collection) were cultivated in Dulbecco's modified Eagle's medium (D5796; Sigma-Aldrich, St. Louis, MO) supplemented with 10% fetal bovine serum (S1760; Biowest, Nuaillé, France). PEIpro (no. 101000026; Polyplus transfection) was used for transfection with pAAV-CMV-ZsGreen1 (SYN6339; Takara Bio, Inc., Kusatsu, Japan), pHelper (SYN6340; Takara Bio, Inc.), and *rep-cap* plasmid (pRC1; SYN63341-2; Takara Bio, Inc.) for rAAV1; pRC2mi342 (SYN6341-1; Takara Bio, Inc.) for rAAV2; or pRC5 (A101-1; Takara Bio, Inc.) on 293T cells in the HYPER*Flask* cell culture vessel (no. 10034; Corning, NY) (1:1:1 ratio).

The cell density reached 70–90% confluence at the time of transfection. The PEIpro (no. 115; Polyplus Transfection, Illkirch, France)/DNA weight ratio was maintained at 1:1 in serum-free Dulbecco's modified Eagle's medium. After 72 h post-transfection, rAAV was harvested using the Triton X-100 buffer (0.5% Triton X-100 [no. 1.08643.1000; Merck, Rahway, NJ]) and 2 mM of MgCl_2_ in phosphate-buffered saline (PBS; 10010-049; Thermo Fisher Scientific, Waltham, MA). Lysis buffer was added to the cells, and the cell lysate was incubated for 1 h at 37°C.

The volume of crude cell lysate was decreased (to 1/8 volume) using tangential flow filtration (N02-E100-05-N; Spectrum, Stamford, CT) and applied to a HiTrap AVB Sepharose Column (28-4112-12; GE Healthcare). rAAV was eluted with 50 mM glycine-HCl (pH 2.7) after washing. Further purification of rAAV was performed by CsCl density gradient centrifugation at 148,500 × g for 46 h at 21°C. Full rAAV particles were then applied to dialysis against PBS and stored at −80°C until use.

### Determination of host cell DNA in rAAV preparations and titration

1908_rAAV1-CMV-ZsGreen1, 1909_rAAV2-CMV-ZsGreen1, and 1910_rAAV5-CMV-ZsGreen1 were treated with or without 1 unit of DNase for 30 min at 37°C, and DNase activity was terminated by adding EDTA at a final concentration of 50 mM. The rAAV capsid was denatured by treatment at 95°C for 10 min. The samples were sequentially diluted with TE_PF_ as necessary, and ddPCR was performed to obtain copy numbers of 18S rRNA genes, as described above.

Subsequently, the copy numbers were converted into mass concentration using Equation (1). For titration, rAAV DNA was similarly prepared with DNase treatment, and ddPCR was performed with the primers and probe described above. Cycling conditions were as follows: 10 min at 95°C, followed by 40 cycles of a two-step thermal profile of 30 s at 94°C and 60 s at 60°C.

## RESULTS

### Determination of the 18S rRNA gene copy number in the HEK293 haploid

A common and rapid method of detecting nucleic acids is absorbance measurement at 260 nm.^18,23^ Assays using fluorescent dyes are sensitive and specific for detecting double-stranded or single-stranded nucleic acids. However, these assays do not always yield reproducible DNA concentrations due to contaminated nucleic acids, and fluorescent dye-binding methods yield contradictory results for genomic DNA depending on the type of DNA calibrator or fluorescent dye.^24^

Thus, the gene copy number obtained by ddPCR has been used for quantitating genomic DNA concentration.^20,25^ Duewer et al.^20^ evaluated seven human reference gene targets using ddPCR and converted the copy number per microliter into nanograms of nuclear DNA per microliter.

We used three reference genes for quantitation of genomic DNA extracted from HEK293 cells through ddPCR. The three amplicons in *EIF5B*, *DCK*, and *HBB* were 67, 83, and 76 bp long, respectively (Table 1); the copy numbers of these genes per 1 ng HEK293 genomic DNA were 284 ± 24, 282 ± 13, and 267 ± 3, respectively, as measured using ddPCR. We next determined the copy number of 18S rRNA genes in HEK293 genomic DNA.

**Table 1.** 18S Ribosomal RNA gene copy number in HEK293 genomic DNA

Target gene	Chromosome	Length of amplicon (bp)	Gene copy number/ng HEK293 genomic DNA, mean ± SD (*n* = 6)	Mean	Ratio (18S rRNA/reference gene)
*EIF5B,* NC_000002.12^a^	Chr 2	67	284 ± 24	278	—
*DCK,* NC_000004.12^a^	Chr 4	83	282 ± 13		
*HBB*, NC_000011.10^a^	Chr 11	76	267 ± 3		
18S rRNA (F1-R)^b^	Chr 13, 14, 15, 21, and 22	116	(5.65 ± 0.12) × 10^4^	5.58 × 10^4^	201
18S rRNA (F2-R)^b^		247	(5.52 ± 0.06) × 10^4^		

^a^ Name of the gene and its accession number.

^b^ Primer set.

DCK, deoxycytidine kinase; EIF5B, eukaryotic translation initiation factor 5B; HBB, hemoglobin subunit beta; rRNA, ribosomal RNA.

The human genome contains ∼200 copies of rRNA genes in the nucleolar organizer regions (NORs) on five acrocentric chromosomes.^21^ André et al. established a qPCR method using primers and probes targeting the human 18S rRNA gene to quantitate host cell DNA.^17^ They set two qPCR amplicons (shorter and longer than 200 bp) for 18S rRNA genes to evaluate the presence of DNA fragments longer than 200 bp. The longer amplicon size was 247 bp from 1,587 to 1,833 for forward F2 and reverse R primers, and the shorter amplicon size was 116 bp from 1,718 to 1,833 for forward F1 and reverse R primers (Fig. 1). Sequences of the F1 primer, F2 primer, and probe 1 were the same as those used previously,^17^ and the reverse primer R was designed at a 10-nt 5′-flanking region from the previous reverse primer (1,811–1,833).

**Figure 2.**
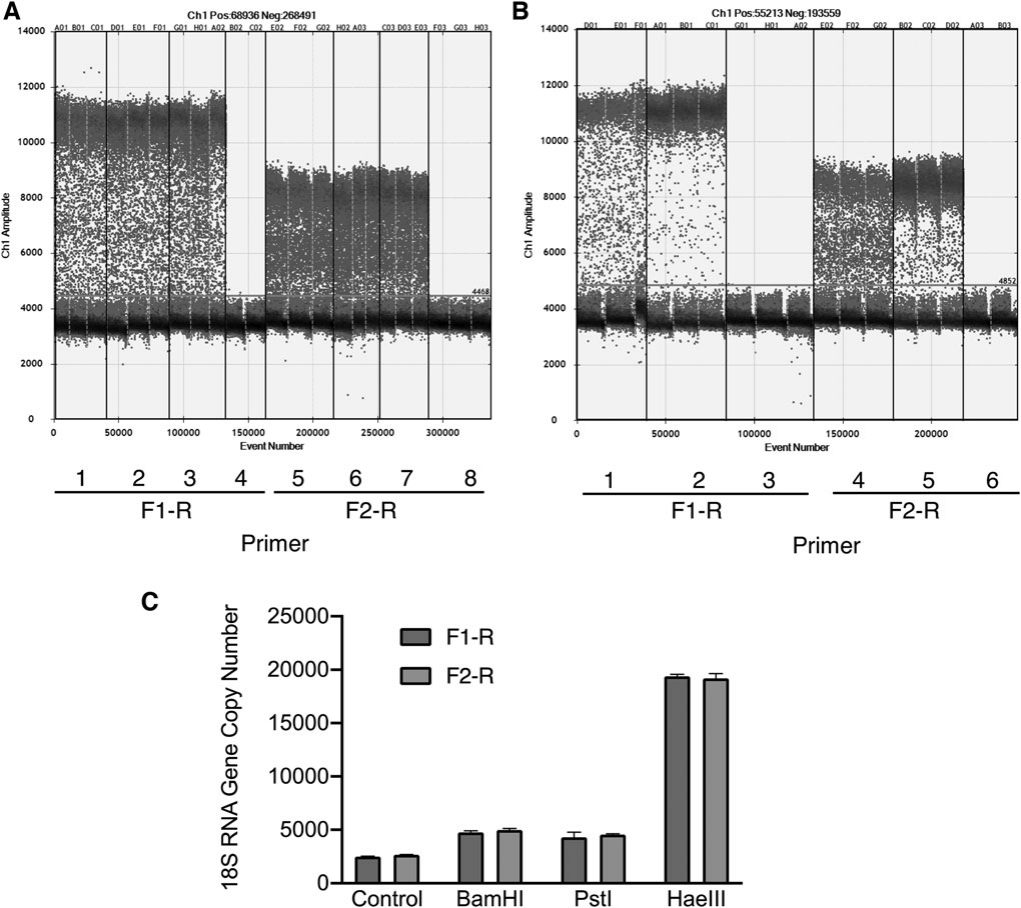
Effect of digestion of HEK293 genomic DNA on ddPCR. **(A)** ddPCR with F1-R primers in 20 μL of reaction mixture, 1, 566 pg of undigested HEK293 genomic DNA; 2, 566 pg of genomic HEK293 DNA digested with *Bam*HI; 3, 566 pg of HEK293 genomic DNA with *Pst*I; and 4, no template. ddPCR with F2-R primers in 20 μL of reaction mixture, 1, 566 pg of undigested HEK293 genomic DNA; 2, 566 pg of HEK293 genomic DNA digested with *Bam*HI; 3, 566 pg of HEK293 genomic DNA with *Pst*I; and 4, no template. **(B)** ddPCR with F1-R primers in 20 μL of reaction mixture, 1, 350 pg of undigested HEK293 genomic DNA; 2, 350 pg of HEK293 genomic DNA digested with *Hae*III; and 3, no template. ddPCR with F2-R primers in 20 μL of reaction mixture, 4, 350 pg of undigested genomic HEK293 DNA; 5, 350 pg of HEK293 genomic DNA digested with *Hae*III; and 6, no template. **(C)** Number of positive droplets in ddPCR with 350 pg of HEK293 genomic DNA with and without digestion by restriction enzymes using F1-R or F2-R primers.

The copy number of 18S rRNA in HEK293 genomic DNA was determined through ddPCR using the F1-R and F2-R primer pairs (Fig. 2A). Positive droplets could not be clearly distinguished from negative droplets without digestion by restriction enzymes (Fig. 2A). Although treatment of genomic DNA with the 6-base cutters, *Bam*HI and *Pst*I, increased the number of positive droplets by approximately twofold, treatment with restriction enzymes failed to improve the profile (Fig. 2B).

Digestion with the 4-base cutter, *Hae*III, allowed clear discrimination of positive droplets from negative droplets, and the copy number of the 18S rRNA gene increased by up to 8.5-fold (Fig. 2C). *Hae*III digestion could generate a 403-bp DNA fragment from the 18S rRNA gene (M10098.1), and the amount of template used in ddPCR revealed a linear relationship (*r*^2^ = 1) with the copy number of 18S rRNA DNA (data not shown).

The copy numbers obtained using F1-R and F2-R primer combinations were 5.65 × 10^4^ and 5.52 × 10^4^ per 1 ng HEK293 genomic DNA, respectively, indicating that the mean copy number was 5.58 × 10^4^ copies per ng (Table 1). The mean copy number per ng of *EIF5B*, *DCK*, and *HBB* was 278. These results indicate that the HEK293 haploid contained 201 copies of the 18S rRNA gene.

### Determination of the limit of detection and limit of quantitation

The limit of detection (LOD) of 18S rRNA DNA for the 118- and 247-bp amplicons in HEK293 genomic DNA was determined (each run containing three to four trials) (Supplementary Table S1). A series of *Hae*III-digested HEK293 genomic DNA dilutions was analyzed at concentrations ranging from 0.7 to 0.0875 ng/mL. The F1-R primer pair could detect the 18S rRNA gene at concentrations as low as 0.0875 ng/mL in 11 of 11 tests, and the F2-R primer pair detected the gene in 9 of 10 tests. The LOD for the 18S rRNA gene was determined at the lowest amount of genomic DNA, with a >95% detection rate. LODs were 0.0875 and 0.175 ng/mL for the F1-R and F2-R primer pairs, respectively.

The limit of quantitation (LOQ) was determined using a similar series of diluted templates with three tests (Supplementary Table S2). The criterion for the LOQ was ≤25% CV. The lowest amount of genomic DNA quantified with a CV of ≤25% was 0.875 ng/mL for the F1-R and F2-R primer pairs.

### Recovery of 18S rRNA from spiked HEK293 genomic DNA

To determine the recovery rate of host cell DNA in the presence of rAAV, we spiked HEK293 genomic DNA into rAAV samples (Table 2). DNA (140 pg per reaction) was added to the rAAV preparations, and the copy number of the 18S rRNA gene was determined by ddPCR. We determined the total amount of human genomic DNA with or without 140 pg of spiked HEK293 DNA.

**Table 2.** Recovery of 18S ribosomal RNA gene copy number in HEK293 DNA-spiked recombinant adeno-associated virus

rAAV^a^	Primer pair (size of the amplicon)	Spiked HEK293 genomic DNA (pg/reaction)^a^	HEK293 genomic DNA (pg/reaction)^b^	Recovery (%)
1908_rAAV1-CMV-ZsGreen1	F1-R (116 bp)	0	108 ± 0	97.9
		140	245 ± 3	
	F2-R (247 bp)	0	86 ± 2	92.1
		140	215 ± 1	
1909 _rAAV2-CMV-ZsGreen1	F1-R (116 bp)	0	36 ± 2	92.9
		140	166 ± 3	
	F2-R (247 bp)	0	27 ± 1	88.6
		140	151 ± 3	
1910 _rAAV5-CMV-ZsGreen1	F1-R (116 bp)	0	117 ± 3	96.4
		140	252 ± 4	
	F2-R (247 bp)	0	82.0 ± 4	91.4
		140	210 ± 2	

^a^ Purified HEK293 DNA was used for spiking experiments. With or without addition of 140 pg DNA into 20 μL of the reaction mixture, the total amount of HEK293 genomic DNA was determined.

^b^ ddPCR was performed in triplicate, and the copy numbers of 18S rRNA were converted into mass concentration of HEK293 genomic DNA using Equation (1). Mean ± SD (*n* = 3).

rAAV, recombinant adeno-associated virus.

In 1908_rAAV1-CMV-ZsGreen1, addition of 140 pg host cell DNA resulted in a total concentration of 245 pg/reaction host cell DNA determined using the F1-R primer pair, while 108 pg/reaction host cell DNA was detected without spiking when using the F1-R primer pair, indicating that 97.9% of HEK293 DNA was recovered. Similarly, 92.9% and 96.4% of spiked DNA could be recovered in 1909_rAAV2-CMV-ZsGreen1 and 1910_rAAV5-CMV-ZsGreen1.

The recovery rate obtained using the F2-R primer pair for the four preparations was 88.6–92.1%. These results support the application of these assays for detection of human cell DNA based on 18S rRNA gene copies.

### Quantitation of HEK293 genomic DNA using F1-R and F2-R primer pairs

The ddPCR-based assay was applied to quantitate host cell DNA in the rAAV preparations (Table 3). Viral titers of 1908_rAAV1-CMV-ZsGreen1, 1909_rAAV2-CMV-ZsGreen1, and 1910_rAAV5-CMV-ZsGreen1 were 1.5 × 10^13^, 1.2 × 10^13^, and 1.6 × 10^13^ vg/mL, respectively. In 1908_rAAV1-CMV-ZsGreen1, the amounts of host cell DNA were 7.41 ± 0.05 and 6.26 ± 0.11 pg/10^9^ vg for the 116- and 247-bp amplicons in the 18S rRNA gene. These results indicated that 85% of ≥116-bp 18S rRNA DNA are ≥247 bp long.

**Table 3.** Quantitation of host cell DNA in recombinant adeno-associated virus samples by droplet digital polymerase chain reaction

rAAV	Titer (vg/mL)^a^	DNase treatment	116 bp (F1-R)		DNase treatment	247 bp (F2-R)		Ratio (247 bp/116 bp
			(pg/10^9^ vg)^b^	DNase-resistant 116 bp (%)		(pg/10^9^ vg)^c^	DNase-resistant 247 bp (%)	
1908_rAAV1-CMV-ZsGreen1	1.5 × 10^13^	−	7.41 ± 0.05	67.3	−	6.26 ± 0.11	58.9	0.85
		+	4.99 ± 0.15		+	3.69 ± 0.11		0.74
1909_rAAV2-CMV-ZsGreen1	1.2 × 10^13^	−	2.67 ± 0.12	64.4	−	1.98 ± 0.06	69.2	0.74
		+	1.72 ± 0.08		+	1.37 ± 0.04		0.78
1910_rAAV5-CMV-ZsGreen1	1.6 × 10^13^	−	6.91 ± 0.11	69.3	−	5.07 ± 0.11	68.2	0.73
		+	4.79 ± 0.18		+	3.46 ± 0.18		0.72

^a,b,c^ Mean ± SD (*n* = 3).

DNase treatment of the rAAV failed to decrease the concentration of contaminated host cell DNA, and no considerable fragmentation was observed from the 247-bp amplicon into the 116-bp amplicon. After DNase I treatment of the rAAV at 37°C for 30 min, 67.3% of the 116-bp amplicon and 58.9% of the 247-bp amplicon were still detected, and the ratio of 247-bp to 116-bp amplicons modestly decreased from 0.85 to 0.74. Similar results were also obtained in 1909_rAAV2-CMV-ZsGreen1 and 1910_rAAV5-CMV-ZsGreen1.

Without DNase treatment, 1909_rAAV2-CMV-ZsGreen1 contained 2.67 ± 0.12 pg/10^9^ vg for the 116-bp amplicon and 1.98 ± 0.06 pg/10^9^ vg for the 247-bp amplicon, and the ratio of 247- to 116-bp amplicons was 0.74. The DNase-resistant fractions of these host cell DNA were 64.4% for the 116-bp amplicon and 69.2% for the 247-bp amplicon, and the ratio of 247- to 116-bp amplicons was 0.78.

In addition, 1910_rAAV5-CMV-ZsGreen1 contained 6.91 ± 0.11 pg/10^9^ vg for the 116-bp amplicon and 5.07 ± 0.11 pg/10^9^ vg for the 247-bp amplicon without DNase treatment. The contaminated host cell DNA contained a DNase-resistant fraction of 68.2–69.3%, and similar fragmentation rates of 0.73 and 0.72 were observed with and without DNase treatment, respectively. Similar data were obtained by qPCR, as shown in Supplementary Table S3.

## DISCUSSION

Previous studies have determined the contamination level of host cell DNA in purified rAAV preparations using qPCR-based methods^8,14,16–18,26–28^ and high-throughput sequencing.^29^ André et al.^17^ described a qPCR-based method to assess the degradation of host cell DNA for preparing vaccine products using two amplicon sizes targeting 18S rRNA DNA. The risk of oncogenicity and infection associated with host cell DNA can be decreased by reducing the size of the DNA to below that of a functional gene.

In this study, we established a ddPCR-based assay to determine the amount of host cell DNA using 116- and 247-bp amplicons. For obtaining reliable results, ddPCR should be performed using intact and accessible targets, and the samples must be free of PCR inhibitors. ddPCR directly estimates the number of positive droplets after partitioning of DNA templates; thus, multiple amplicons should not be present in the same strands, and each template must be independently distributed to the droplets. These conditions were satisfied for the 18S rRNA gene, but not for the Alu sequences (data not shown).

Human diploid cells contain ∼400 copies of a 43-kb ribosomal DNA unit containing the 18S rRNA gene tandemly arrayed in NORs on five acrocentric chromosomes.^30,31^ Thus, digestion of HEK293 genomic DNA with *Hae*III was required for facilitating the independent distribution of the 18S rRNA template (Fig. 2).

Residual DNA impurities represent a safety concern in rAAV production. Hauck et al.^8^ quantitated 6.3–208 pg/10^9^ vg of DNA in the rAAV purified by gen1 chromatography or chromatography gradient methods using a qPCR-based method with 18S rRNA genes and they found the maximum size of residual host cell DNA to be 4,300 nucleotides through Southern blot analysis with a ^32^P-labeled HEK293 genomic DNA probe. Ayuso et al.^16^ detected 32–461 pg/10^9^ vg host cell DNA in rAAV prepared using a two-time CsCl gradient centrifugation protocol.

In this study, we detected 2.67–7.41 pg/10^9^ vg DNA for the 116-bp amplicon and 1.98–6.26 pg/10^9^ vg DNA for the 247-bp amplicon using ddPCR without DNase treatment (Table 3). The use of two amplicon sizes revealed that rAAV preparations contained similar DNase-resistant fractions (64.4–69.3% for the 116-bp amplicon and 58.9–69.2% for the 247-bp amplicon) (Table 3). The guidelines recommend reducing the amount of free residual DNA to less than 10 ng per dose and the DNA size to below ∼200 bp.^15,32^

Taking into consideration the high dose of rAAV (*e.g.,* 1.1 × 10^14^ vg/kg for Zolgensma), the recommended value may be challenging to achieve because of encapsulation of DNA impurities, including host cell DNA.^26^ Although the precise mechanisms underlying host cell DNA packaging into AAV particles remain unknown,^8,9^ host cell DNA may also be unintentionally packaged by Rep through interaction with a preferential binding sequence (such as a Rep-binding element).

In the case of DNA impurities acquired from plasmids used for transfection, preferential packaging of plasmid DNA fragments containing the P5 promoter sequence has been reported.^33^ These studies suggest that DNA impurities may be packaged into capsids in a single-stranded form.^9^ Elucidating the form of host cell DNA packaged into capsids and preferential packaging is necessary even though single-stranded DNA may not have a risk similar to that of double-stranded DNA.

## Supplementary Material

Supplemental data

Supplemental data

Supplemental data
