# Supplementary material for: Quantitation of Residual Host Cell DNA in Recombinant Adeno-Associated Virus Using Droplet Digital Polymerase Chain Reaction
Source: Hum Gene Ther. 2023 Jun 16;34(11-12):578–85. doi: 10.1089/hum.2023.006 (PMC10285681; doi:10.1089/hum.2023.006)
Supplement: Supplemental data [file Suppl_TableS1.docx]

**Supplemental Table 1. Limit of detection for 18S rRNA gene**

| Primer pair  (Size of amplicon) | HEK293 genomic DNA ^a^ (pg/reaction) | test #1 positive/run | test #2 positive/run | test #3 positive/run | positive /total runs | Positive rate^b^ (%) |
| --- | --- | --- | --- | --- | --- | --- |
| F1-R (116 bp) | 0.7 | 4/4 | 4/4 | 3/3 | 11/11 | 100 |
|  | 0.35 | 3/3 | 4/4 | 4/4 | 11/11 | 100 |
|  | 0.175 | 3/3 | 3/3 | 3/3 | 9/9 | 100 |
|  | 0.0875 | 4/4 | 4/4 | 3/3 | 11/11 | 100 |
|  | 0 | 0/4 | 0/4 | 0/3 | 0/11 | 0 |
| F2-R (247 bp) | 0.7 | 4/4 | 4/4 | 3/3 | 11/11 | 100 |
|  | 0.35 | 4/4 | 4/4 | 4/4 | 12/12 | 100 |
|  | 0.175 | 4/4 | 4/4 | 4/4 | 12/12 | 100 |
|  | 0.0875 | 2/2 | 3/4 | 4/4 | 9/10 | 90 |
|  | 0 | 0/4 | 0/4 | 0/3 | 0/11 | 0 |

^a^ Concentration of HEK293 genomic DNA digested with HaeIII was independently determined by conversion of mean copy number of the three reference genes assayed by ddPCR. The DNA template was diluted with TE_PF_ to 0.7, 0.35, 0.175, 0.0875 ng/ml.

^b^LOD is defined as at lowest concentration of the template with 100% positive rate.
