# Supplementary material for: Quantitation of Residual Host Cell DNA in Recombinant Adeno-Associated Virus Using Droplet Digital Polymerase Chain Reaction
Source: Hum Gene Ther. 2023 Jun 16;34(11-12):578–85. doi: 10.1089/hum.2023.006 (PMC10285681; doi:10.1089/hum.2023.006)
Supplement: Supplemental data [file Suppl_TableS2.docx]

**Supplemental Table 2. Limit of quantification for 18S rRNA gene using**

| Primer pair  (Size of amplicon) | HEK293 genomic DNA^a^ (pg/reaction) | mean^a^ (copies/reaction) | SD^b^ | CV^c^ (%) |
| --- | --- | --- | --- | --- |
| F1-R (116 bp) | 14 | 737 | 30 | 4.1 |
|  | 7 | 373 | 22 | 6.0 |
|  | 3.5 | 186 | 15.7 | 8.4 |
|  | 1.75 | 94.9 | 6.3 | 6.7 |
|  | 0.875 | 47.1 | 8.2 | 17.5 |
|  | 0 | 0 | 0 | – |
| F2-R (247 bp) | 14 | 781.6 | 34.9 | 4.5 |
|  | 7 | 378.4 | 12.6 | 3.3 |
|  | 3.5 | 192.7 | 13.9 | 7.2 |
|  | 1.75 | 98.9 | 13.9 | 14.0 |
|  | 0.875 | 51.0 | 1.7 | 3.4 |
|  | 0 | 0.2 | 0.3 | – |

^a^ Concentration of HEK293 genomic DNA digested with HaeIII was independently determined by conversion of mean copy number of the three reference genes assayed by ddPCR. The DNA template was diluted with TE to 0.7, 0.35, 0.175, 0.0875 ng/ml. ddPCR was performed three times in triplicate (n = 9).

^b^SD, standard deviation.

^c^CV, coefficient of variation.
