# Supplementary material for: Quantitation of Residual Host Cell DNA in Recombinant Adeno-Associated Virus Using Droplet Digital Polymerase Chain Reaction
Source: Hum Gene Ther. 2023 Jun 16;34(11-12):578–85. doi: 10.1089/hum.2023.006 (PMC10285681; doi:10.1089/hum.2023.006)
Supplement: Supplemental data [file Suppl_TableS3.docx]

**Supplemental Table 3. Quantitation of host cell DNA in rAAV samples by qPCR^a^**

| rAAV |  | 116 bp (F1-R) | |  |  | 247 bp (F2-R) | | Ratio  (247 bp/116 bp |
| --- | --- | --- | --- | --- | --- | --- | --- | --- |
|  | DNase  treatment | (pg/10^9^ vg) ^b^ | DNase-resistant  116 bp  (%) |  | DNase  treatment | (pg/10^9^ vg)^c^ | DNase-resistant  247 bp  (%) |  |
| 1908_rAAV1-CMV-ZsGreen1 | − | 6.05 ± 0.31 | 83.6 |  | − | 4.49 ± 0.31 | 96.0 | 0.74 |
|  | + | 5.06 ± 0.85 |  |  | + | 4.31 ± 0.21 |  | 0.85 |
| 1909_rAAV2-CMV-ZsGreen1 | − | 2.00 ± 0.12 | 84.5 |  | − | 1.31 ± 0.03 | 87.8 | 0.66 |
|  | + | 1.69 ± 0.16 |  |  | + | 1.15 ± 0.13 |  | 0.68 |
| 1910_rAAV5-CMV-ZsGreen1 | − | 5.30 ± 0.11 | 94.5 |  | − | 3.51 ± 0.56 | 98.3 | 0.66 |
|  | + | 5.01 ± 0.18 |  |  | + | 3.45 ± 0.57 |  | 0.69 |

^a^ qPCR was performed using the same primer pairs and the probe with QuantiTect Probe PCR Kit (cat#204343); 95˚C for 15 min followed 40 cycles of denaturation and anneling/extension.

^b,c^Mean ± SD (n = 3)
